# Sequence variability of BamA and FadL candidate vaccinogens suggests divergent evolutionary paths of *Treponema pallidum* outer membrane proteins

**DOI:** 10.1128/jb.00159-25

**Published:** 2025-07-14

**Authors:** Everton B. Bettin, Farhang Aghakhanian, Christopher M. Hennelly, Wentao Chen, Timothy C. Davenport, Simon T. Hackl, Andre A. Grassmann, Fabio Vargas-Cely, Sebastián Silva, Jonny A. García-Luna, Lady G. Ramirez, Yinbo Jiang, Ligang Yang, Heping Zheng, Bin Yang, Petra Pospíšilová, David Šmajs, Mitch M. Matoga, Irving F. Hoffman, Eduardo López-Medina, Kay Nieselt, M. Anthony Moody, Arlene C. Seña, Juan C. Salazar, Jonathan B. Parr, Melissa J. Caimano, Kelly L. Hawley, Justin D. Radolf

**Affiliations:** 1 UConn Health 705913, Farmington, Connecticut, USA; 2 The University of North Carolina at Chapel Hill 2331, Chapel Hill, North Carolina, USA; 3 Dermatology Hospital, Southern Medical University 70570, Guangzhou, Guangdong, China; 4 Department of Molecular Genetics and Microbiology, Duke University School of Medicine 12277 , Durham, North Carolina, USA; 5 Institute for Bioinformatics and Medical Informatics, University of Tübingen 9188, Tübingen, Baden-Württemberg, Germany; 6 Division of Hematology and Medical Oncology, Mayo Clinic 23386, Phoenix, Arizona, USA; 7 Centro Internacional de Entrenamiento e Investigaciones Médicas (CIDEIM) 439458, Pance, Valle del Cauca, Colombia; 8 Universidad Icesi 28012, Cali, Valle del Cauca, Colombia; 9 Czech Republic Department of Biology, Faculty of Medicine, Masaryk University 37748, Brno, South Moravian Region, Czechia; 10 UNC Project-Malawi 156288 , Lilongwe, Central Region, Malawi; 11 Universidad del Valle 28006, Cali, Valle del Cauca, Colombia; 12 Duke University Medical Center 609772, Durham, North Carolina, USA; 13 Connecticut Children's 718903 , Hartford, Connecticut, USA; National Institutes of Health, Bethesda, Maryland, USA

**Keywords:** syphilis, outer membrane proteins, protein variability, whole-genome sequencing, vaccines

## Abstract

Knowledge of *Treponema pallidum* subspecies *pallidum* (*TPA*) outer membrane protein (OMP) sequence variability is essential for understanding spirochete proliferation within endemic populations as well as the design of a globally effective syphilis vaccine. Our group has identified extracellular loops (ECLs) of *TPA* BamA (TP0326) and members of the FadL family (TP0548, TP0856, TP0858, TP0859, and TP0865) as potential components of a multivalent vaccine cocktail. As part of a consortium to explore *TPA* strain diversity, we mapped the variability of BamA and FadL orthologs in 186 *TPA* strains from Malawi, China, and Colombia onto predicted 3D structures. The 186 genomes fell into eight subclades (five Nichols- and three SS14-lineage) with substantial geographic restriction. Single nucleotide variants accounted for the large majority of proteoforms, with variability notably higher within the Nichols-lineage strains. Most mutations were in regions of the proteins predicted to be extracellular and harboring B cell epitopes. We observed a striking difference in the degree of variability between the six OMPs, suggesting that these proteins are following divergent evolutionary paths. Concatenation of OMP sequences recapitulated the phylogenetic structure of the *TPA* strains, effectively segregating within clades and largely clustering by subclades. Finally, we noted that BamA and FadL candidate ECL vaccinogens, previously shown to elicit antibodies that kill treponemes during *in vitro* cultivation, are well conserved. Taken as a whole, our study establishes a structural-phylogenetic approach for analyzing the forces shaping the host-pathogen interface in syphilis within endemic populations while informing the selection of vaccine targets.

**IMPORTANCE:**

Syphilis remains a major global health concern, reinforcing the need for a safe and effective vaccine. Understanding the variability of *TPA* OMPs is essential for tracking pathogen evolution and informing vaccine design. Here, we analyzed the variability of six *TPA* OMPs in 186 strains from Malawi, China, and Colombia, identifying protein-specific evolutionary patterns. Most mutations were localized in extracellular regions and, notably, appeared to correlate with the phylogenetic structure of *TPA*. Despite OMP heterogeneity, several candidate vaccinogens remained highly conserved, reinforcing their potential as globally effective vaccine targets. Our study establishes a structural-phylogenetic framework for dissecting the forces shaping the host-spirochete interface within endemic populations and provides a foundation for designing a globally effective syphilis vaccine.

## INTRODUCTION

Syphilis is a multistage, sexually transmitted infection (STI) caused by the highly invasive and immunoevasive spirochete *Treponema pallidum* subspecies *pallidum* (*TPA*) (1, 2). The disease has undergone a worldwide resurgence since the start of the new millennium (3); even though its causative agent remains exquisitely susceptible to penicillin after more than seven decades of use (3, 4). These alarming epidemiological trends underscore the need for a safe and effective vaccine as well as an improved understanding of the basic biology and virulence properties that foster proliferation of the syphilis spirochete within at-risk populations (5–7). *TPA* is a diderm bacterium with an unorthodox outer membrane (OM) lacking lipopolysaccharide (LPS) and containing a low density of integral outer membrane proteins (OMPs) and a paucity of surface-exposed lipoproteins (5, 8–10). The molecular architecture of the spirochete’s OM is the ultrastructural basis for its impressive capacity to evade innate and adaptive host defenses, giving rise to its designation as the “stealth pathogen” (5, 10, 11). Despite the lack of sequence similarity between *TPA* OMPs and their counterparts in gram-negative bacteria, advances in protein three-dimensional (3D) structure prediction have enabled the identification of transmembrane β-barrel-forming proteins encoded within the *TPA* genome (10, 12). *TPA*’s repertoire of OMPs (the *TPA* “OMPeome”) includes two stand-alone proteins, BamA and LptD, involved in OM biogenesis and four paralogous families involved in importation of nutrients or extrusion of noxious substances across the OM: 8-stranded β-barrels, OM factors for efflux pumps, *TPA* repeat proteins, and orthologs for FadL long-chain fatty acid transporters (10, 13).

*TPA* is widely considered to be an extracellular bacterium (5, 14, 15, 19). To affect spirochete clearance, antibodies elicited during infection or by vaccination must target extracellular regions of the bacterium’s OMPs (5–7). In recent years, we have identified surface-exposed regions in BamA and in members of the FadL family as candidate components of an experimental syphilis vaccine cocktail (16–19). BamA (TP0326) is the central component of the molecular machinery that inserts newly exported OMPs into the spirochete’s OM (20). Like its gram-negative counterparts, BamA in *TPA* is bipartite and consists of a predicted 16-stranded β-barrel with eight extracellular loops (ECLs) and five polypeptide transport-associated (POTRA) domains within the periplasm (Fig. S1A) (13, 17, 21). The five *TPA* FadL-like orthologs (TP0548, TP0856, TP0858, TP0859, and TP0865) contain structural features found in canonical fatty acid transporters: a 14-stranded β-barrel with seven ECLs and an N-terminal hatch domain that plugs the lumen of the barrel (Fig. S1A) (22, 23). Unlike canonical FadLs, however, the hatches of the *TPA* orthologs are predicted to extend into the extracellular milieu, where they should be antibody accessible (13). Three *TPA* FadLs (TP0548, TP0859, and TP0865) also contain unique C-terminal tetratricopeptide (TPR) domains predicted to reside in the periplasmic compartment (13, 24).

Knowledge of *TPA* OMP variability is a prerequisite for understanding spirochete proliferation within endemic regions as well as the design of a globally efficacious syphilis vaccine. While advancements in whole-genome sequencing (WGS) of *TPA* strains in clinical specimens have significantly expanded our knowledge of *TPA* genetic diversity (25–34), these analyses generally have not focused on OMP sequence variability. Moreover, publicly available *TPA* genomic sequences usually represent a limited number of strains from different geographic locations as opposed to deep sampling at individual sites. Herein, as part of a global consortium to explore *TPA* strain diversity in the context of syphilis vaccine design (31), we sequenced 186 *TPA* strains from our clinical research sites in Malawi, China, and Colombia, and subsequently mapped the variability of BamA and the five FadL orthologs onto their predicted 3D structures. Most mutations were in regions predicted to be extracellular and harboring B cell epitopes (BCEs), suggesting that host immune pressure is a major driver of OMP diversity. A striking difference in the degree of variability between the six OMPs was observed, suggesting they are following divergent evolutionary paths. The OMP profiles generated for each strain within our cohort recapitulated the phylogenetic structure of *TPA*, segregating by clades and largely by subclades. These findings indicate that recombination is not a major factor underlying variation of this portion of the OMPeome, and they raise the possibility that pressures arising from demographic or regional factors contribute to OMP evolution. Finally, we noted a high degree of conservation among BamA and FadL candidate ECL vaccinogens that elicit antibodies capable of killing treponemes during *in vitro* cultivation (18, 19). Taken as a whole, our study establishes a structural-phylogenetic approach for analyzing the forces shaping the host-spirochete interface within endemic populations. Our study also informs the selection of surface-exposed targets for a vaccine that could eliminate a disease that has afflicted humankind for centuries.

## RESULTS

### Sources and phylogenies of *TPA* genomes

**Fig 1:**
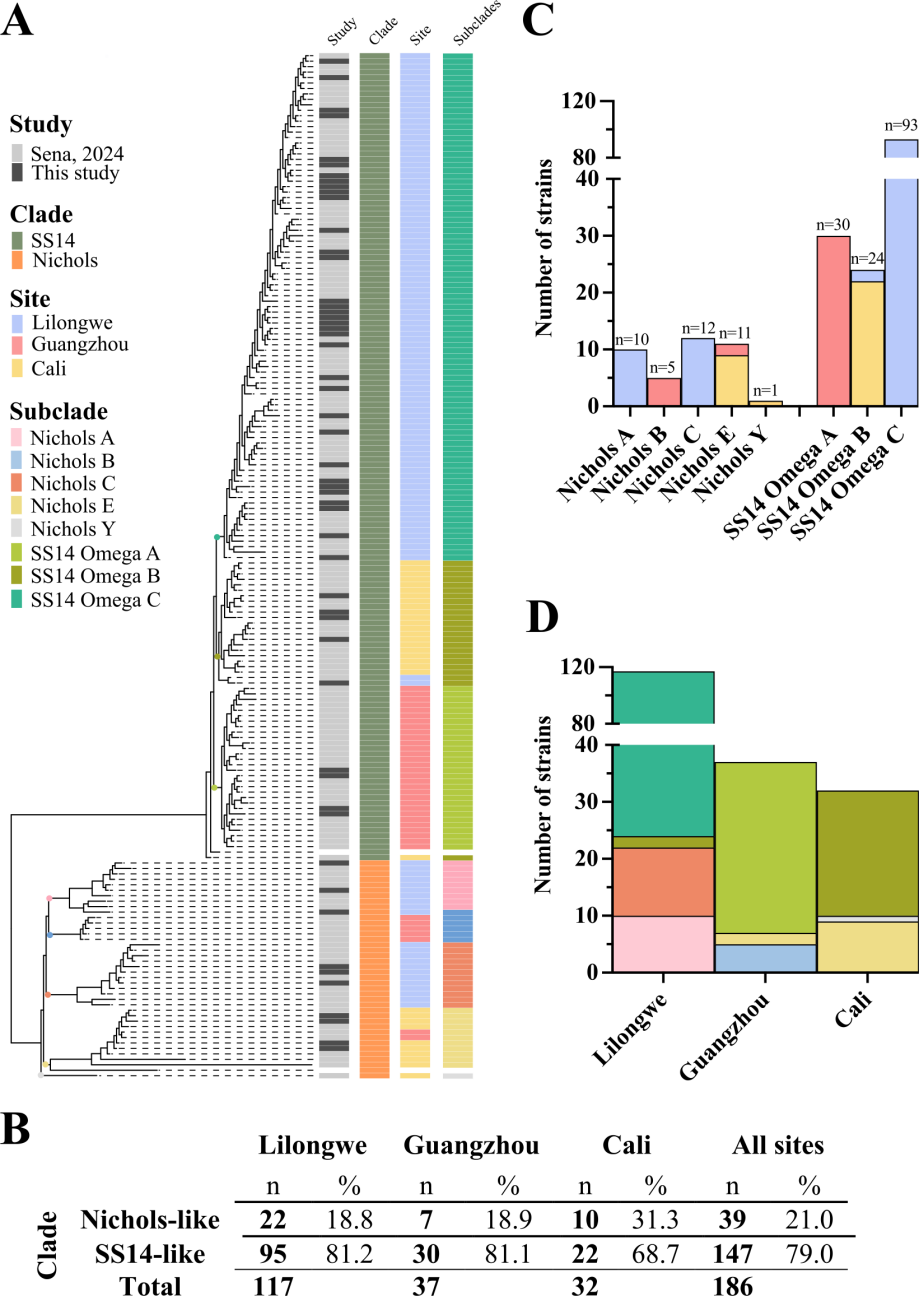
Distribution of the 186 strains within our cohort according to their subclades and sites. (**A**) Recombination-masked *TPA* whole-genome phylogeny demonstrated that SS14-lineage strains were predominant within our cohort. Strains were assigned to eight distinct *TPA* subclades: five Nichols (Nichols A, B, C, E, and Y) and three SS14 (SS14 Omega A, B, and C). (**B**) Similar proportions of Nichols- and SS14-lineage strains were found in Lilongwe and Guangzhou (~81.1%), but a lower (68.7%) in Cali. (**C and D**) All *TPA* subclades exhibited a high degree of geographic restriction.

To explore *TPA* strain diversity in the context of syphilis vaccine design (31), we recruited individuals with early syphilis (primary, secondary, and early latent syphilis) at clinical research sites in Lilongwe, Malawi; Guangzhou, China; and in Cali, Colombia. Genomic sequences from *TPA* strains infecting 131 patients were recently reported (31). For the present study, we added 55 unique genomes from all three sites (Fig. 1A), yielding a total of 186 genomic sequences obtained between 2015 and 2022 (see Table S1 for strain information). Detailed demographic information about subjects included in this study is available in our previous publications (31, 32, 35).

While the majority (~78.5%) of strains were directly sequenced from genital ulcer swabs or secondary syphilis skin biopsies, 37 genomic sequences from Guangzhou and 3 from Cali were obtained after *TPA* passage in rabbits (Table S1). We have previously confirmed that rabbit passage has minimal effects on *TPA* genomic stability and does not introduce mutations in the analyzed loci (32). In agreement with other recent studies (25–28, 33), phylogenomic analysis revealed a predominance (79%; *n* = 147) of SS14-lineage strains within our cohort (Fig. 1A and B). The proportion of SS14-lineage strains was virtually identical (~81%) in Lilongwe and Guangzhou, but lower (68%) in Cali (Fig. 1B); of note, a higher prevalence of Nichols-lineage strains has been observed in other genomic surveys from South America (26, 28).

To facilitate comparison with previous genomic analyses (31, 36), the strains were curated based on maximum likelihood phylogeny (Fig. 1A), resulting in five Nichols (Nichols A, B, C, E and Y) and three SS14 (SS14 Omega A, B and C) subclades. The subclade formerly called SS14 Omega East-Asia (31, 36) herein was renamed SS14 Omega A. The remaining SS14-lineage strains, previously grouped as a single SS14 Omega subclade (31), formed two distinct branches on the tree, and, therefore, were classified as the SS14 Omega B and C subclades (Fig. 1A). All subclades exhibited a substantial degree of geographic restriction (Fig. 1C), with only one lineage in each clade (Nichols E and SS14 Omega B) containing a small number of strains from another site. Also noteworthy is that different combinations of Nichols and SS14 subclades were co-circulating in each site. All three sites contained strains from two Nichols subclades (Fig. 1D), while Lilongwe, which provided the most strains to the cohort, was the only site containing strains from two different SS14 subclades (Omega B and C).

### *TPA* BamA and FadL 3D models

Using a Biopython-based computational framework developed by our group, we identified mutations in the BamA and FadL sequences within our cohort and mapped them onto 3D structures generated by AlphaFold3 (37), a state-of-the-art deep learning tool for protein structure prediction. Note that throughout this study, we refer to individual BamA and FadL variants as “proteoforms.” All β-strand residues exhibited high confidence values at a minimum, as defined by AlphaFold3, supporting accurate delineation of the boundaries between β-strands and ECLs (Fig. S1). Notably, in the AlphaFold3 model for BamA, ECL3 is longer and ECL4 is shorter than previously predicted by the ModWeb server, which used the *Neisseria gonorrhoeae* BamA (PDB ID: 4K3B) as a template (17) (Fig. S2). AlphaFold3 models for the FadLs aligned closely with those previously generated using trRosetta (13). Both algorithms predict that the *TPA* FadLs lack a β-strand three kink, a characteristic feature of canonical FadLs (23, 38), and have hatches that extend into the extracellular milieu, suggesting that *TPA* orthologs may use alternative mechanisms for substrate capture and transport.

### Substitutions in BamA occurred exclusively in ECLs and largely segregated with subclades

**Fig 2:**
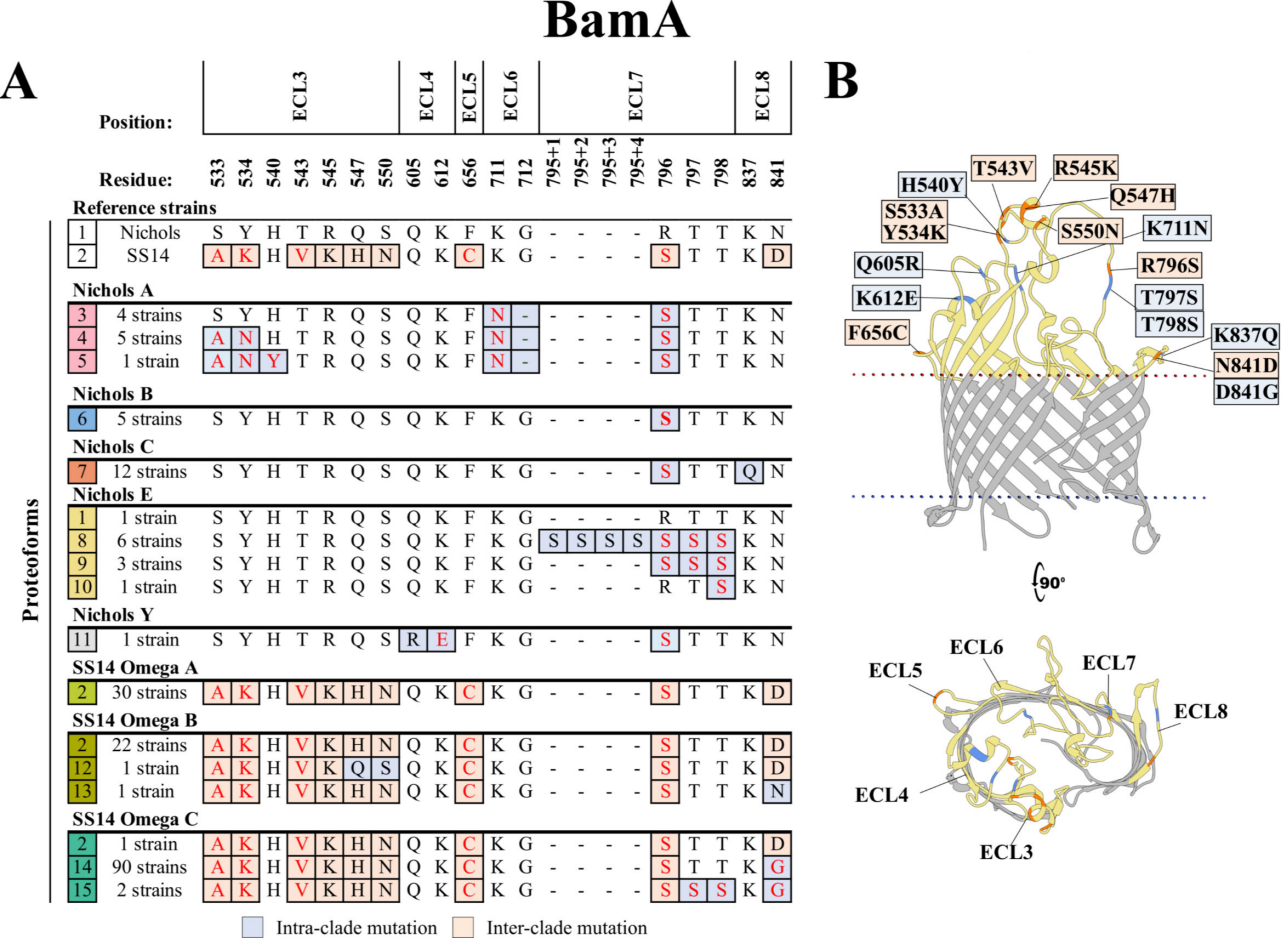
Substitutions in BamA are exclusively in ECLs and largely segregate with *TPA* subclades. (**A**) Amino acid residue differences between the 15 identified BamA proteoforms (1–15 in the leftmost squares). Nine “discriminator” residues (peach boxes) distinguish the proteoforms in Nichols and SS14 reference strains. We identified 17 additional intra-clade mutations (gray-blue boxes) in ECLs. Non-conservative substitutions are colored in red. Residues are numbered according to their position in the Nichols reference sequence. (**B**) Amino acid substitutions were mapped onto the AlphaFold3 model for the Nichols reference BamA β-barrel. Periplasmic POTRA domains are hidden in the model to facilitate visualization.

BamA in the Nichols and SS14 reference strains (designated proteoforms 1 and 2, respectively) differ by nine residues, with six of these mutations in ECL3 (Fig. 2). Substitutions in five of these nine reference strain “discriminators” are non-conservative, as determined by Grantham physicochemical distance (39). Three of the reference strain discriminators (T543, R545, and F656 in the Nichols reference) were conserved throughout the cohort. We identified nine Nichols variants containing 15 intra-clade mutations (proteoforms 3–11) and 4 SS14 variants with six intra-clade mutations (proteoforms 12–15); most replacements arose from single nucleotide variants (SNVs) (Table S2). Only 1 of the 39 Nichols-lineage strains (Nichols E from Cali) contained a BamA identical to the Nichols reference. In contrast, 53 of the 147 strains, found predominantly in the SS14 Omega A and B subclades contained exact matches to the SS14 reference sequence. All mutations were located exclusively in ECLs and largely segregated with subclades (e.g., variable ECLs 3 and 6 in Nichols A, ECL 7 in Nichols E, and ECL 8 in SS14 Omega B and C). The vast majority (92 out of 94) of the SS14-lineage strains in our cohort with a variant BamA were in the Omega C subclade and contained a D841G mutation in ECL8 (proteoform 14).

### TP0856 and TP0859 are highly conserved

**Fig 3:**
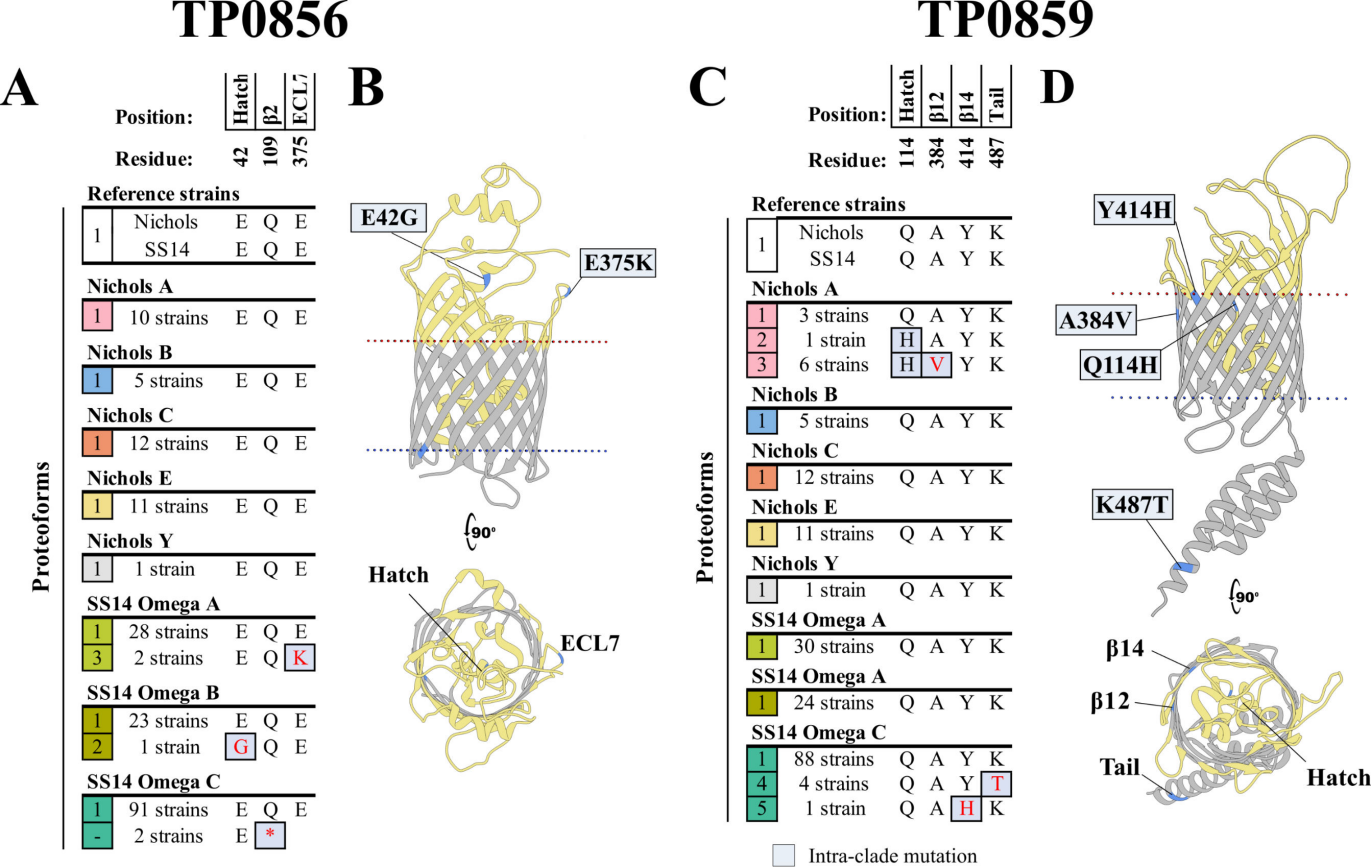
TP0856 and TP0859 are highly conserved. TP0856 and TP0859 proteoforms from Nichols and SS14 reference strains are identical. (**A**) Amino acid residue differences between the three identified TP0856 proteoforms (1-3 in the leftmost squares). Only three SS14-lineage strains contained TP0856 proteoforms with intra-clade mutations (gray-blue boxes). Two *tp0856* sequences contained an early stop codon at position 327. (**B**) Predicted mutations in ECL7 and the hatch of TP0856 variants from Guangzhou (SS14 Omega A) and Cali (Omega B). (**C**) Amino acid residue differences between the five identified TP0859 proteoforms (1–5 in the leftmost squares). Intra-clade mutations were identified in seven Nichols-lineage and five SS14-lineage strains. (**D**) Predicted mutations in the hatch, β-strands, and periplasmic regions of TP0859 variants. A portion of the hatch is hidden in the model to facilitate visualization. In (**A **and **C),** non-conservative substitutions are colored in red. Residues are numbered according to their position in the Nichols reference sequence.

The TP0856 sequences are each identical in Nichols and SS14 reference strains (proteoform 1) are identical. No mutations in TP0856 were observed in the 39 Nichols-lineage strains in our cohort (Fig. 3A and B). Two SS14 Omega A strains from Guangzhou and one Omega B strain from Cali contained SNVs leading to non-conservative substitutions in ECL7 and hatch (proteoforms 2 and 3), respectively (Fig. 3A and B; Table S2). Notably, two SS14 Omega C strains from Lilongwe harbored *tp0856* alleles containing a nonsense SNV at position 327 (Fig. 3A and B; Table S2). TP0856 was the only OMP analyzed herein in which more variants were found within the SS14-lineage. As with TP0856, the Nichols and SS14 reference sequences for TP0859 are identical (proteoform 1) (Fig. 3C and D). SNVs generated four TP0859 variant proteoforms in a small number of strains (seven Nichols A and five Omega C) from Lilongwe.

### TP0858 variants result primarily from intra-clade mutations but also a rare recombination

**Fig 4:**
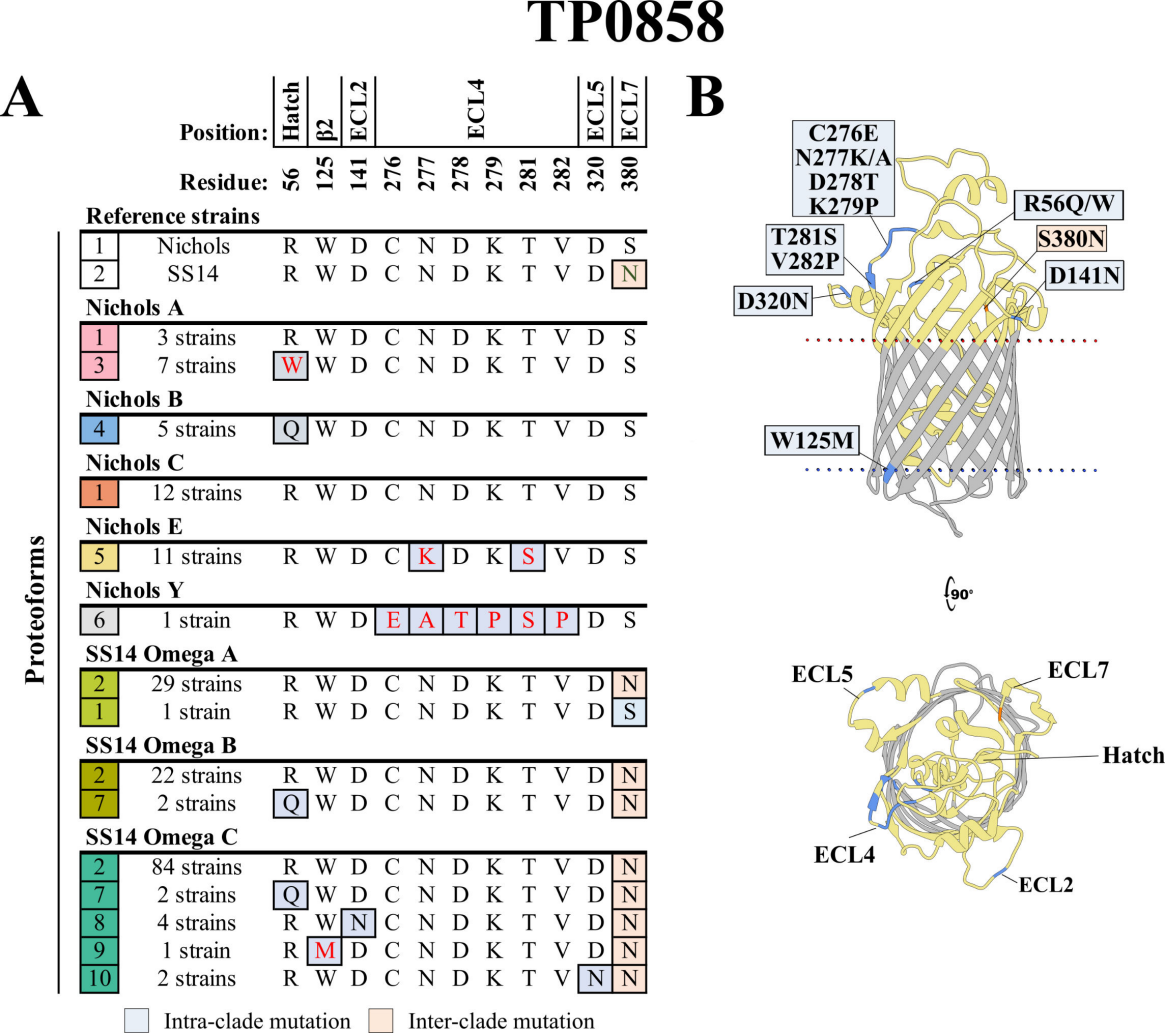
Amino acid substitutions in TP0858 result primarily from intra-clade mutations but also a rare recombination. (**A**) Amino acid residue differences between the 10 identified TP0858 proteoforms (1–10 in the leftmost squares). The TP0858 sequences in the Nichols and SS14 reference genomes differ by only an S380N substitution (peach boxes). Eight variant proteoforms containing intra-clade mutation (gray-blue boxes) were identified within the cohort. All 11 Nichols E strains carried mutations (N277K and T281S) in ECL4. In the sole Nichols Y strain, a putative recombination between ECL4 of TP0856 and TP0858 was identified within residues 276–282 of ECL4. Non-conservative substitutions are colored in red. Residues are numbered according to their position in the Nichols reference sequence. (**B**) Substitutions mapped onto the AlphaFold3 model for the Nichols reference.

A single, conservative amino acid substitution due to an SNV in ECL7 (S380N) distinguishes the TP0858 reference sequences in the Nichols and SS14 (proteoforms 1 and 2; Fig. 4; Table S2). Intra-clade substitutions generated by SNVs at seven different positions, along with a recombination in one strain, gave rise to four TP0858 variants in each clade (proteoforms 3–6 and 7–10, respectively). More than half (24 out of 39) of the Nichols-lineage strains contained TP0858 variants compared to only 12 of the 147 SS14-lineage strains. The sole Nichols Y strain contained a TP0858 variant (proteoform 6) with a previously reported (40, 41) recombination in ECL4 that replaces residues 276–282 with the corresponding residues (271–277) of TP0856. Interestingly, one SS14 Omega A strain harbored the Nichols reference due to an SNV that converted the single SS14 reference discriminator asparagine at position 380 to a serine residue.

### TP0548 is hypervariable due to Nichols and SS14 clade-specific substitutions within the hatch and ECL2, respectively

**Fig 5:**
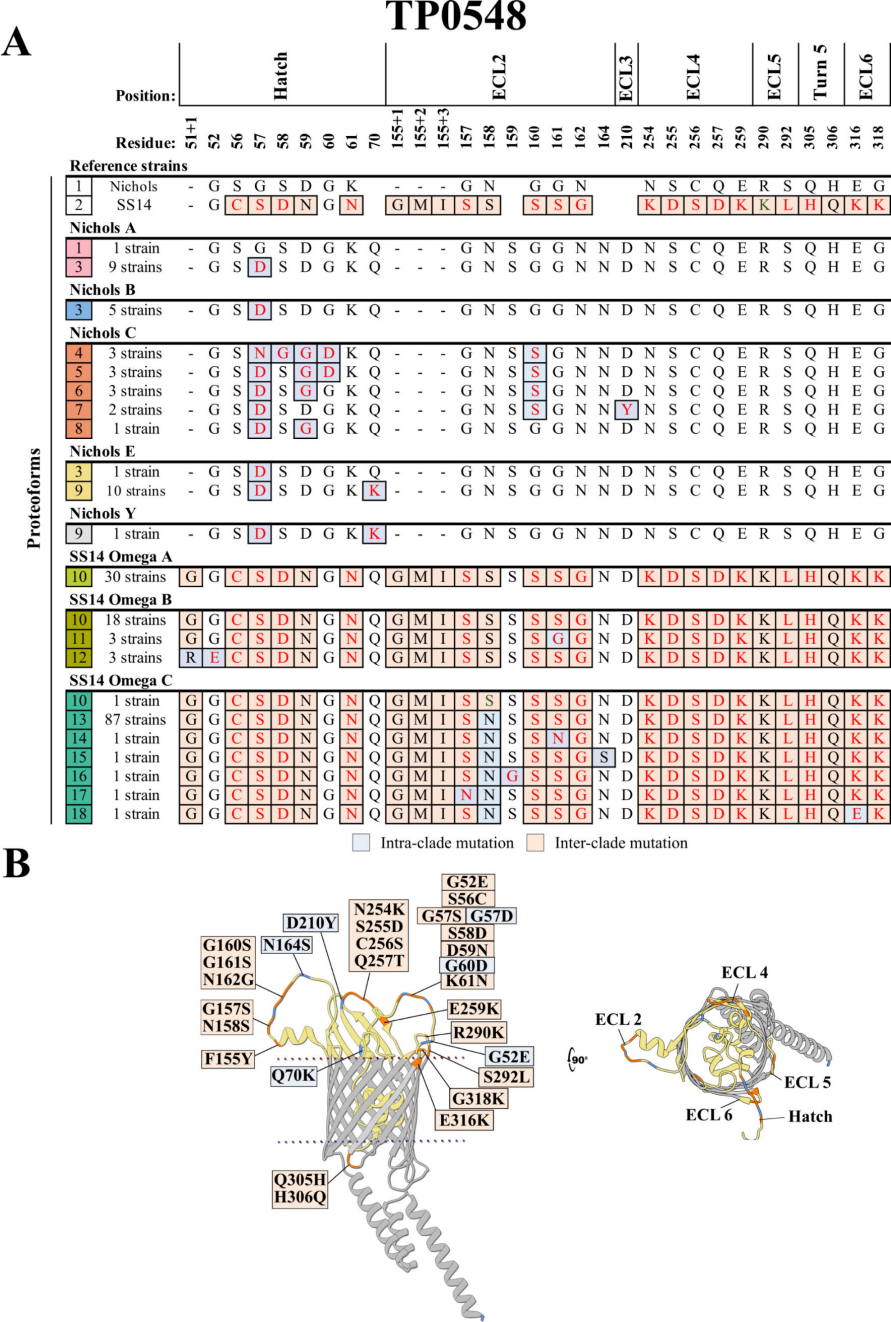
TP0548 is hypervariable with Nichols- and SS14-specific substitutions within the hatch and ECL2, respectively. (**A**) Amino acid residue differences between the 18 identified TP0548 proteoforms (1–18 in the leftmost squares). Reference sequences for Nichols and SS14 contain 25 inter-clade mutations (peach boxes). We identified 16 variant proteoforms within our cohort, eight in each clade, containing intra-clade substitutions (gray-blue boxes) at 15 amino acid positions. Most of the intra-clade differences are found in the hatch and in ECL2, for the Nichols-lineage and SS14-lineage strains, respectively. Non-conservative substitutions are colored in red. Residues are numbered according to their position in the Nichols reference sequence. (**B**) Substitutions were mapped onto the AlphaFold3 model for the Nichols reference.

The Nichols and SS14 TP0548 reference sequences (proteoforms 1 and 2) differ by 22 substitutions (17 non-conservative) along with a three amino acid insertion in ECL2 of the SS14 reference OMP (Fig. 5). We identified a total of 16 variants containing intra-clade mutations generated by SNVs in 15 positions, eight at reference strain discriminators (Fig. 5; Table S2). Within the Nichols clade, only one Nichols A strain (from Lilongwe) contained a sequence identical to the clade reference. A striking feature of TP0548 variants within the Nichols lineage (proteoforms 3–9) was the predominance of intra-clade substitutions predicted to reside in the extracellular portion of the hatch. Among the 147 SS14-lineage strains, there were no exact matches to the reference due to a ubiquitous glycine insertion after position 51 of the hatch. A variant defined exclusively by this insertion (proteoform 10) was found in all 30 SS14 Omega A strains and in 18 of the 24 Omega B strains, but in only one Omega C strain. Intriguingly, residues of the hatch that were variable within the Nichols clade were highly conserved across SS14-lineage strains. However, 92 of the SS14 Omega C strains contained substitutions within five closely spaced residues in ECL2 (proteoforms 13–18).

### TP0865 in the Nichols E subclade is hypervariable due to an inter-subspecies recombination

**Fig 6:**
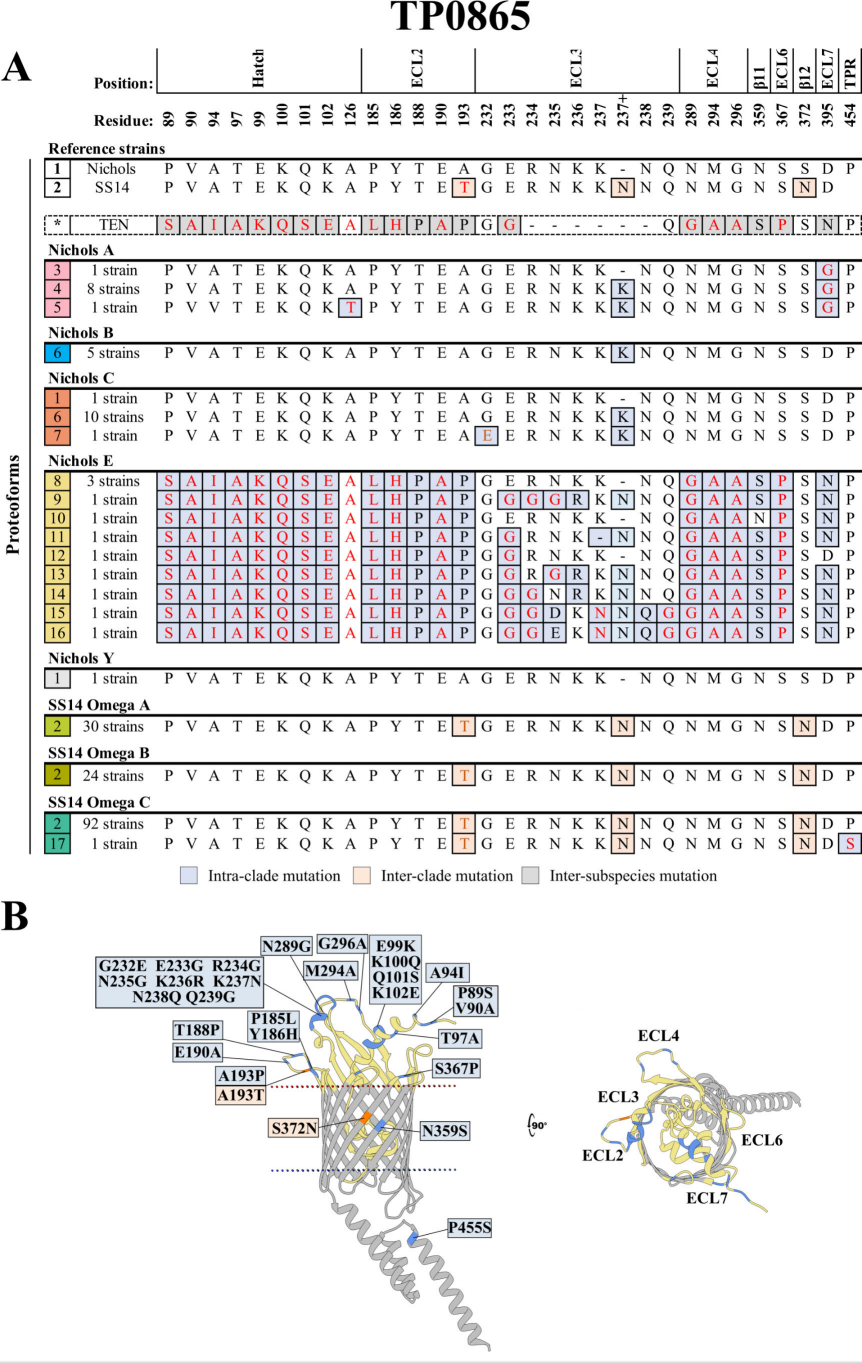
TP0865 in the Nichols E subclade contains an ancient inter-subspecies recombination and a hypervariable ECL3. (**A**) Amino acid residues differences between the 17 identified TP0865 proteoforms (1–17 in the leftmost squares). Three inter-clade mutations (peach boxes) distinguish the Nichols and SS14 references. We identified 15 variant TP0865 proteoforms containing intra-clade mutations (gray-blue boxes); nine variants were within the Nichols E subclade. The high variability observed within Nichols E aligns with the previous report of recombination events between *TPA* and endemicum treponemes, as evidenced by the inter-subspecies substitutions (gray boxes) in *Treponema endemicum* (*TEN*) TP0865 proteoform (*). In contrast, TP0865 was highly conserved among SS14-lineage strains. Non-conservative substitutions are colored in red. Residues are numbered according to their position in the Nichols reference sequence. (**B**) Substitutions mapped onto the AlphaFold model for the Nichols reference. A portion of the hatch is hidden in the model to facilitate visualization.

The Nichols and SS14 TP0865 reference sequences (proteoforms 1 and 2) differ by non-conservative and conservative amino acid substitutions originating, respectively, from SNVs within ECL2 (A193T) and β-strand 12 (S372N) along with the insertion of an asparagine after position 237 in ECL3 of the SS14 reference (Fig. 6; Table S2). Only one reference strain discriminator (S372N) was conserved within all Nichols-lineage strains, while the SS14-lineage strains contained no substitutions in discriminators. Only 2 of the 39 Nichols-lineage strains (Nichols C, Lilongwe and Nichols Y, Cali) contained TP0865 sequences identical to the clade reference. We identified five variants (proteoforms 3–7) within the Nichols A–C subclades containing substitutions at four positions within the hatch, ECL3, and ECL7. By contrast, the Nichols E subclade contained nine variants (proteoforms 8–16) harboring substitutions at 27 positions predominantly in the hatch and ECLs 2–4. The majority (19 out of 27) of the substitutions were in two stretches of DNA encoding residues 89–193 and 289–395 (i.e., flanking ECL3) previously reported to have arisen by recombination between *TPA* and endemicum treponemes (*TEN/TPE*) (42–44) (Fig. S3); an additional eight substitutions generated hypervariability in the nonrecombinant stretch encoding ECL3. In stark contrast, only one of the 147 SS14-lineage strains (Omega C, Lilongwe) contained a non-conservative substitution (P454S) in the periplasmic TPR domain (proteoform 17).

### OMP profiles reflect divergent OMP evolution within clades and subclades

**Fig 7:**
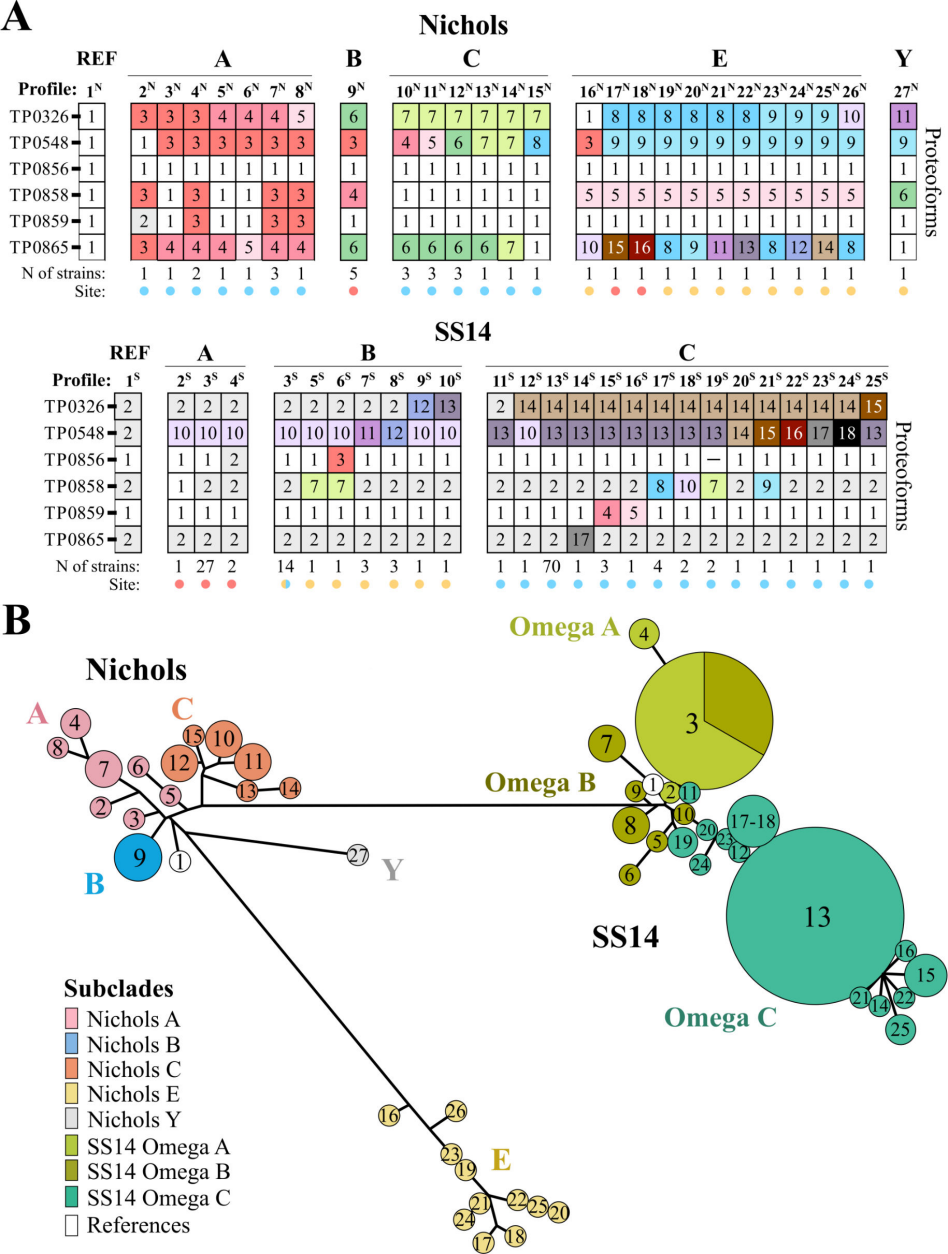
OMP profiles reflect divergent OMP evolution within clades and subclades. (**A**) OMP profiles (columns) were created by concatenating proteoforms (numbered boxes) for all six OMPs (rows) in each strain. Also shown are the number (N) of strains sharing a profile and their respective sites (colored dots: Lilongwe, light blue; Guangzhou, salmon; and Cali, yellow). (**B**) Minimum spanning tree (MST) for concatenated sequences was created using the stand-alone GrapeTree package with the neighbor-joining algorithm. Clusters are labeled with the proteoform numbers assigned in (A). Strain IDs and their corresponding profiles are listed in Table S3.

We next sought to ascertain how OMP variability within our cohort correlated with the phylogenetic structure of *TPA*. We began by concatenating proteoforms for all six OMPs in each strain to generate OMP profiles (Fig. 7A; Table S3). Intriguingly, no strain within our cohort contained a profile identical to the Nichols and SS14 clade references (profiles 1^N^ and 1^S^, respectively). We identified 26 profiles among the 39 Nichols-lineage strains (profiles 2^N^–27^N^) compared to 24 within the 147 SS14-lineage strains (profiles 2^S^–25^S^). Strikingly, no OMP profiles were shared between Nichols subclades, while 27 of the 30 SS14 Omega A and 14 of the 24 SS14 Omega B strains shared profile 3^S^. Nichols B was the only subclade in which all five strains (from Guangzhou) contained the same profile (profile 9^N^). In a number of instances, variability of profiles within a clade can be attributed to SNVs in individual OMPs. For example, OMP profiles within the Nichols C strains contained a variable TP0548 mainly due to substitutions in the hatch, while hypervariability in ECL3 of TP0865 accounted for the high number of profiles within the Nichols E subclade. Within the SS14 clade, SNVs in ECL2 of TP0548 largely accounted for the divergent profiles within Omega C strains.

To further elucidate the relationships between OMP profiles, we next generated OMP-based minimum spanning trees (MSTs) using the GrapeTree stand-alone tool (45) (Fig. 7B). The MSTs underscored that OMP profiles segregated strictly by clade, and largely by subclade, as well as the greater degree of variability within the Nichols clade. It was noteworthy that the Nichols reference profile fell on a branch separate from the other Nichols profiles, whereas the SS14 reference clustered closer with the Omega A and B profiles. The divergence of the profiles in the single Nichols Y strain and in all the Nichols E strains was mainly due to the recombinant stretches in TP0858 and TP0865, respectively. A striking difference between clades was the predominance of two profiles (3^S^ and 13^S^) within the SS14-lineage strains, whereas no predominant profile was observed within Nichols-lineage strains. The single glycine insertion in the hatch of TP0548 separated profile 3^S^ from the SS14 reference.

### Variable and conserved surface-exposed regions of *TPA* BamA and FadLs contain predicted BCEs

**Fig 8:**
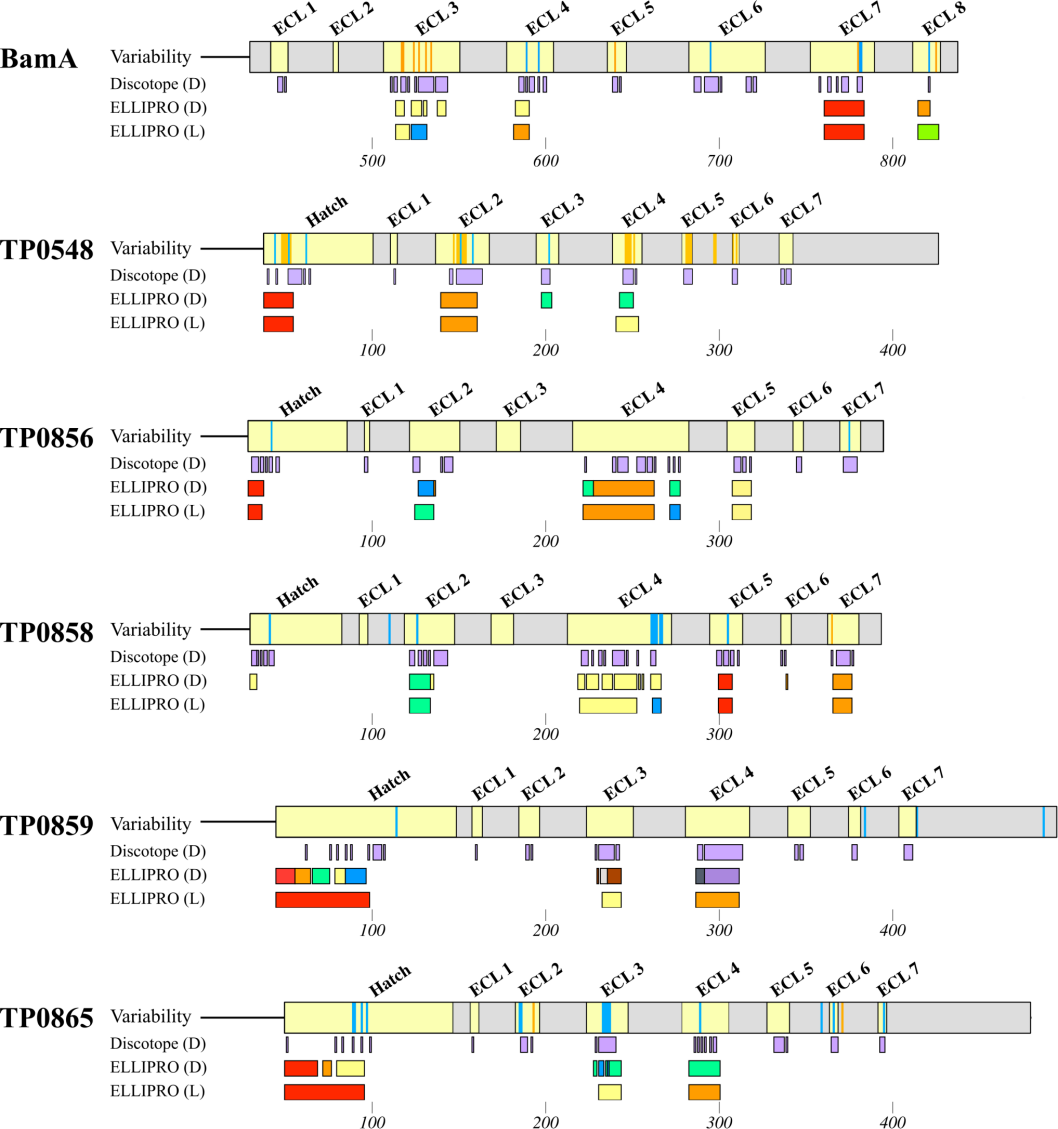
Conserved and variable regions of *TPA* OMPs contain predicted B-cell epitopes. DiscoTope and ELLIPRO servers predict Discontinuous (D) and Linear (L) B-cell epitopes (BCEs) for hatch and ECL regions (yellow) in all six analyzed OMPs. The position of inter-clade (orange stripes) and intra-clade (blue stripes) mutations correlates with predicted BCEs. BCEs were also predicted for conserved surface-exposed regions. Individual epitopes predicted by ELLIPRO were distinguished using different colors; DiscoTope does not differentiate individual epitopes. Graphical representations were generated using MacVector.

As a starting point for assessing the role of immune pressure in driving evolution of OMPs in *TPA*, we used the DiscoTope (46) and ElliPro servers (47) to correlate the position of predicted BCEs with the mutations identified in the six OMPs analyzed herein. As shown in Fig. 8, the vast majority of the variability occurred within regions containing predicted BCEs. A BCE is predicted for the region in BamA ECL3 (533–550) and for the hatch and ECLs in TP0548 where high inter- and intra-clade variability was found. The surface-exposed regions in TP0858, most notably ECLs 2 and 4, with intra-clade mutations, also contained predicted BCEs. Also noteworthy were the BCE predictions for TP0865 in the hypervariable ECL3. Interestingly, the algorithms also made strong epitope predictions for more conserved ECLs in BamA (ECL4) and TP0856 (ECLs 2 and 4) known to be immunogenic (17, 18, 48). Additionally, several regions (hatch, ECL3, and ECL4) of the highly conserved TP0859 also contained predicted BCEs.

### Conservation of candidate ECL vaccinogens

Vaccine design in syphilis is moving toward ECL-based immunization strategies to circumvent the many difficulties inherent in the large-scale production of full-length OMP vaccinogens (7). From our studies, five ECLs (ECL4 from BamA and ECLs 2 and 4 from TP0856 and TP0858) have garnered attention as promising vaccine targets (16–19). The degree of conservation of these immunogenic ECLs, therefore, is a critical determinant of their global efficacy in a vaccine cocktail formulation (7). The Nichols and SS14 reference sequences for all five candidate ECL vaccinogens are identical, and only 16 of the 186 strains in our cohort contained mutations in one or more of the five ECLs (Table 1). BamA ECL4 (one variable strain) and TP0856 ECLs 2 and 4 (no variable strains) were highly conserved. On the other hand, candidate ECL vaccinogens from TP0858 displayed some diversity, with four SS14 Omega C strains harboring a conservative mutation in ECL2 (D141N), all 11 Nichols E strains harboring non-conservative substitutions (N277K and T281S) in ECL4, and the Nichols Y strain containing a recombination in the same ECL. Other surface-exposed regions, such as the TP0856 hatch and ECL3 and ECL4 of TP0859, contain predicted BCEs and were also highly conserved within our cohort (Fig. 8), suggesting they could serve as future targets for vaccine development (Table 1).

**TABLE 1:** Conservation of candidate ECL vaccinogens within our cohort

	Number of strains		Description	
	Conserved	Variable	Mutation	Subclade
BamA ECL4	185	1	Q605R, K612E	Nichols Y
TP0856 ECL2	184	0	N/A^*a*^	N/A
TP0856 ECL4	184	0	N/A	N/A
TP0858 ECL2	182	4	D141N	SS14 Omega C
TP0858 ECL4	174	11	N277K, T281S	Nichols E
		1	Recombination	Nichols Y
TP0856 Hatch	183	1	E42G	Omega B
TP0859 ECL3	186	0	N/A	N/A
TP0859 ECL4	186	0	N/A	N/A

^*a*^ N/A: Not applicable.

## DISCUSSION

In addition to guiding vaccine development, the characterization of an expanded *TPA* OMPeome provides new insights into how syphilis spirochetes adapt to geographically and demographically diverse at-risk human populations. As with other pathogens (49, 50), the need to evade the host immune response likely drives *TPA* OMP adaptation, while the extent to which OMPs can adapt by sequence variation is limited by structural and/or functional constraints. The non-canonical features of *TPA* OMPs introduce additional complexity to understanding this interplay of pressures. Our syphilis vaccine consortium described a large-scale genomic analysis of *TPA* strains (31), pointing out the existence of population-specific differences in ECLs of two *TPA* FadLs (TP0858 and TP0865). In the present study, we expanded this analysis to investigate proteoforms for six OMPs across seven geographically structured *TPA* subclades circulating in three geographically diverse sites. We confirmed that variable residues were predominantly located in surface-exposed regions, often correlating with predicted BCEs. The identification of variable (e.g., BamA and TP0548) and conserved (e.g., TP0856 and TP0859) OMPs brought to light an unexpectedly uneven distribution of evolutionary pressures across this portion of the OMPeome. The similarity in OMP profiles within geographically restricted subclades suggests that regional demographic factors contribute to *TPA* OMP variability.

Recombination is a major driver of OMP diversity in bacteria, occurring both within genomes (intragenomic) and between organisms (intergenomic) (51, 52). In *TPA*, the largest and most diverse OMP familythe *TPA* repeat proteins—exhibits strong evidence of both (53–55). Intragenomic rearrangements also were reported between TP0856 and TP0858 (43). In a previous study of *TPA* clinical isolates (56), we found evidence of intergenomic recombination in BamA, where several Nichols- and SS14-lineage strains harbored BamA variants matching those from other *TPA* lineages, such as Mexico A. Notably, WGS of the Mexico A strain revealed that its BamA variant contained sequences derived from *T. pallidum* subspecies *pertenue* (*TPE*) (57). Recent studies (44, 58) indicate that most recombination events involving BamA and FadLs occurred between ancestors of the *TPA* lineage and endemic treponemes (*TPE/TEN*). In the present study, recombination among *TPA* strains was uncommon, although footprints of previously described ancient intersubspecies events were discernible. For example, recombination between a Nichols E ancestor and *TPE*/*TEN* (44, 58) likely generated the highly variable TP0865 proteoforms observed in all strains within this subclade. Similarly, sequences for BamA and TP0548 were highly divergent between Nichols- and SS14-lineage strains, aligning with ancient intersubspecies recombination involving ancestors of single *TPA* clades (44, 58). In contrast, only a single recent recombination event, a modular rearrangement previously reported for this OMP (43), was identified in a TP0858 proteoform from the Nichols Y strain. The paucity (43) of recent recombination events also suggests that the TP0858 Nichols reference proteoform identified in an SS14 Omega A strain is likely the result of an SNV rather than intergenomic recombination. Intergenomic recombination requires co-infection, which might have been more common when ancient events of genetic exchange occurred among *T. pallidum* subspecies (44, 58). Evidence for co-infection has been reported in the modern era for *TPE* (59), but surprisingly, not for *TPA* (see Authors note) (32, 44, 58). Our analysis, as a whole, suggests that recombination involving *TPA* OMPs is rare, despite the co-circulation of Nichols and SS14 strains across all three sites, and highlights the need for broader sampling to capture these rare events.

SNVs are an important source of variability within OMPs and can significantly alter the virulence properties of bacteria, including their ability to evade host immune responses (60–66). As a stand-alone OMP (13, 21), *TPA* BamA must adapt to immune pressure while maintaining its essential function in OM biogenesis (67). The predicted BCEs for the variable regions in BamA ECL3 and ECL7 would suggest that these are the primary loops under immune pressure. However, ECL4, which was highly conserved within our cohort, also appears to be subject to immune pressure. Previously, we noted that a large proportion of clinical strains from Cali, Colombia contained a substitution (L589Q) in this loop (56) that markedly diminished reactivity with ECL4 antibodies from patients infected with *TPA* strains containing the Nichols reference BamA (56). Antibodies against ECL4 of *Escherichia coli* BamA freeze the BAM complex in the open conformation, preventing insertion of newly synthesized OMPs into the OM bilayer (68, 69); antibodies against *TPA* BamA ECL4 are bactericidal (18, 19), presumably via the same mechanism. The absence of the L589Q substitution in the present study was surprising. It is tempting to speculate that ECL4 antibodies in patients infected with *TPA* strains harboring the glutamine at position 589 in BamA conferred a selective advantage in an endemic population in which L589 predominated.

Similar to BamA, SNVs accounted for the majority of *TPA* FadL variants but with a complex pattern across the family. While TP0856 and TP0859 were highly conserved, the other FadL orthologs displayed considerable variability, with TP0548 containing the highest number of proteoforms. Additionally, the specific ECLs exhibiting the greatest variability differed for each protein: ECL4 in TP0858, ECL2 in TP0548, and ECL3 in TP0865. *TPA* FadL orthologs feature a hatch with an extended region that, as with the ECLs, differed in their degree of variability. Variability in the hatch was particularly pronounced in TP0548 but also present in TP0858 and, to a lesser extent, in TP0856 and TP0859. The variability in some of the *TPA* FadL hatches is consistent with the AlphaFold3 predictions that these regions are surface-exposed, and therefore, likely under immune pressure. In multi-gene families, functional redundancy often enables one gene to adapt while another is constrained to an essential function (70, 71). Notably, the two highly conserved FadLs, TP0856 and TP0859, belong to structurally different FadL subgroups, each containing a more sequence-divergent paralog (13). Further detailed characterization of the individual members of this family will be needed to clarify the structural factors, functional redundancies, and immunological properties driving their divergent evolutionary trajectories. While immunological pressures undoubtedly are a principal force shaping *TPA* FadL variability, we have uncovered evidence that these proteins do not fully conform to the well-established paradigm that variability within bacterial antigens correlates with antigenicity (72, 73). ECLs 2 and 4 of the most conserved FadL (TP0856) are among the most antigenic loops during rabbit and human syphilitic infection (18, 48). Why these ECLs are sequence invariant despite ostensibly intense immunologic pressure is an intriguing question. Calculations of *TPA*’s molecular clock typically are based on genomic sequences from which some OMPs, including FadLs, have been excluded (33, 58). The spectrum of variability within the *TPA* FadL family is in accord with the notion that different regions of a bacterial genome can follow distinct molecular clocks (74). As only a portion of the OMPeome was analyzed in our study, a more comprehensive investigation will be needed to fully assess how *TPA* OMP variability reflects broader molecular clock estimates.

A major finding in our study is that the OMP profiles segregated with Nichols- and SS14-lineage strains. Previous reports have called attention to the greater genomic diversity of Nichols-lineage strains compared to those in the SS14 clade (25–28, 33). The greater variability of OMP profiles within the Nichols clade represents a striking example of how OMP diversity in *TPA* mirrors genomic structure. Generation of OMP variants is generally considered an indicator of bacterial adaptability that leads to enhanced fitness in a particular environment (75–78). However, the predominance of the SS14 lineage in all three geographic sites argues that, in the case of *TPA,* greater OMP variability does not necessarily confer a selective advantage. Analysis of *TPA* OMP profiles also revealed that variability generally followed subclade-specific patterns. For example, ECL3 of BamA was hypervariable within Nichols A, while increased variability in ECL7 was observed within Nichols E. The extreme variability of TP0865 ECL3 within the Nichols E subclade, which is flanked by regions affected by ancient recombinant events, provides another example. Similarly, mutations in the TP0548 hatch domain were prevalent in several Nichols subclades, whereas ECL2 was the variable region within SS14 Omega C strains. One possible explanation for these subclade-specific OMP patterns is that they reflect regional demographic and behavioral pressures within endemic human populations, such as differences in sexual networks, degrees of population mobility, and regional variation in host genetics. It is also possible that spontaneous mutations tolerated in one region of an OMP influence the emergence of mutations in other regions, channeling variability in subsequent generations of the same lineage. In a global phylogenetic study of *TPA* strains using a database collection of 726 genomic sequences, Beale et al. (33) reported identical core genomes shared by strains from 14 geographically dispersed countries, suggesting that *TPA* is genetically homogenous. For reasons that are not readily apparent, *TPA* sublineages in our cohort showed a high degree of geographic restriction (31). Moreover, all but one (profile 3^S^) of the 51 OMP profiles were identified in single locations, suggesting that OMP variability in *TPA* conforms to a phylogenetic geographical structure. It is of interest to note that our MST analysis placed the OMP profile of the Nichols reference strain in a divergent branch from other Nichols-lineage strains, whereas the OMP profile of the SS14 reference more closely resembled those of circulating SS14-lineage strains. The contrast between clade prototypes aligns with the conclusion from the global phylogenomic analysis (33) that the Nichols reference strain represents a lineage that may no longer be widely circulating.

The surface-exposed regions of *TPA* OMPs reside at the frontline of the “arms race” (79) between the spirochete, an extracellular pathogen (5, 14, 15, 19), and its obligate human host. Although our study does not, strictly speaking, provide a longitudinal analysis of *TPA* OMP evolution, it offers an extended snapshot of OMP diversity that can be dissected experimentally to better understand how the spirochete ‘wages’ war within human populations. We coined the term stealth pathogenicity to describe the capacity of *TPA* to evade host antibody responses and establish persistent infection in individuals (5, 10, 11). A key question to emerge from our analysis is how OMP variability promotes stealth pathogenicity within endemic populations. Experiments are currently underway to determine how amino acid substitutions affect antigenic and surface reactivity of ECL-specific antibodies; recently developed mutagenesis techniques for *TPA* (19, 80) can also be applied to solve this problem. Results from these experiments may aid in the selection of optimal vaccine targets or support the inclusion of multiple proteoforms to improve coverage in future syphilis vaccine cocktails. Generating protective ECL antibodies has emerged as a major strategy for ending the arms race (16–19). Even variable *TPA* OMPs contain highly conserved ECLs, some of which have been shown to elicit functional antibodies in rabbits and mice (16–19). Recently, we reported that immune sera from rabbits infected with either the Nichols or SS14 strain have a diminished capacity to impair the viability of the opposite clade during *in vitro* cultivation (18, 19). This observation suggests that antibodies that recognize variable ECLs may be important for complete protection and, by extension, that a broadly protective syphilis vaccine may require a “cocktail” of conserved and variable ECLs.

Some limitations to our study should be acknowledged. First, by focusing on a subset of the *TPA* OMPeome, our analysis did not include OMPs that may be essential for understanding the full adaptive landscape of *TPA*. Additionally, our cohort contained strains from only three countries and almost certainly does not fully capture the global diversity of *TPA* strains. Moreover, we do not know whether some *TPA* subclades (e.g., Nichols Y) are truly rare in our study sites or were underrepresented due to unintended sampling biases. Nevertheless, the cardinal strength of our study is the creation of a structural framework for analyzing multiple OMPs, which, combined with deep sampling in three clinical research sites, yielded a high-resolution view of *TPA* OMP diversity within a geographic and evolutionary context. Future efforts will expand this approach to additional OMPs and sites, enabling further clarification of how host pressures at the population level shape the global *TPA* OMPeome.

## MATERIALS AND METHODS

### Study design and sample collection

We conducted a multi-center study to recruit individuals with early syphilis (primary, secondary, and early latent syphilis) who presented at a STI clinic in Lilongwe, Malawi; a provincial dermatology hospital in Guangzhou, China; and in a public healthcare sector network in Cali, Colombia. The recruitment period spanned from November 2019 to May 2022. Details about eligibility criteria for participant enrollment, including obtaining informed consent, are described in Sena et al. (31). Additional information about strains within our cohort is available in Table S1. Patient enrollment and sample collection were approved by the institutional review boards (IRBs) of the Dermatology Hospital of Southern Medical University in Guangzhou (IRB protocol: Guangdong Dermatology Hospital Lunli Shencha-20181202 [R3]); CIDEIM in Cali (IRB 163, protocol 1289); the National Health Sciences Research Committee, Ministry of Health, in Lilongwe (IRB approval 2252); and the University of North Carolina at Chapel Hill (IRB protocol 19-0311).

### WGS and variant calling

Specimens containing 40 or more copies of *TPA polA* per µL underwent *TPA* enrichment followed by WGS (31). Enrichment was performed using parallel, pooled whole-genome amplification (30) and custom 120-nucleotide RNA oligonucleotide baits (Agilent Technologies, Santa Clara, CA, USA). Libraries were generated using either Sure Select XT Low Input or XTHS2 kits, as previously described (31). Pooled enriched libraries subsequently were sequenced using MiSeq or NovaSeq platforms (Illumina, San Diego, CA, USA), employing paired-end sequencing with 150-base pair reads. Sequencing data were analyzed using a pipeline available at https://github.com/IDEELResearch/tpallidum_genomics. Briefly, adapters were removed using Trimmomatic (v0.39) (81), and host genomes were filtered out using bbmap (v38.82) (82) by mapping reads to human (hg19) or rabbit (oryCun2) reference genomes. Sequence alignments were performed with bwa (v0.7.17) (83) against the Nichols (CP004010.2) and SS14 (CP004011.1) reference sequences. Post-alignment, sequences underwent filtering to exclude reads with excessive mismatches, excessive soft or hard clipping, chimeric reads, and low mapping quality. Only sequences with at least 80% coverage by three reads were retained. Variant calling was conducted using GATK HaplotypeCaller (v4.4) (84), and consensus genomes were constructed with GATK FastaAlternateReferenceMaker.

### Phylogenetic analysis

For phylogenetic analysis, we used only SNPs to infer evolutionary relationships. To ensure data reliability, we masked *TPA* genomes for repetitive and difficult-to-sequence genes, including *arp*, *tp470*, and the *tpr* gene cluster. Additionally, we employed Gubbins (v3.2) (85) to identify and remove putative recombination regions. Genome alignments were performed using MAFFT (v4.790) (86) with Nichols (CP004010.2) and SS14 (CP004011.1) as reference genomes. The final phylogenetic tree was constructed with RAxML (v8.2.12) (87) and visualized using R (v4.1.2) (88) with the ggtree package (v3.2.1) (89). Clades and subclades were manually assigned to facilitate comparison with published analysis (31, 36).

### Characterization of BamA and FadL proteoforms

Sequences for BamA and FadL orthologs (TP0548, TP0856, TP0858, TP0859, and TP0865) were extracted, and their proteoforms were further analyzed using a Biopython script available at https://github.com/ebbettin/UCH_SRL. To avoid false variant calls, homopolymeric regions in genomic sequences from clinical strains were manually curated to match the references. Consequences of missense variants were defined using Grantham’s physicochemical distances (39), where substitutions with Grantham differences > 50 were considered non-conservative. Sequences for BamA and FadLs within the same strain were concatenated, and MSTs for concatenated sequences were created using the stand-alone GrapeTree package with the neighbor-joining algorithm (45).

### Structural modeling and B-cell epitope predictions

3D models for *TPA* Nichols reference strain proteome (UP000000811) were generated using AlphaFold3 (37). Protein models were visualized using UCSF Chimera v1.1 (90), and identified mutations were mapped using attribute assignment files generated by the Biopython script described above. To delineate ECL boundaries, the positioning of proteins in the membrane was predicted using the OPM (orientation of proteins in membranes) database (91, 92). Linear and discontinuous epitopes were predicted by ElliPro (47) and DiscoTope 3.0 (46) using cut-off values of 0.7 and 1.5, respectively. Graphical representations of protein sequences were created with MacVector v18.5.

## SUPPLEMENTAL MATERIAL

Supplemental figures jb.00159-25-s0001.pdf Figures S1 to S3.

Table S1 jb.00159-25-s0002.xlsx Description of *TPA* strains included in the study.

Table S2 jb.00159-25-s0003.xlsx Description of identified proteoforms and their respective nucleotide mutations.

Table S3 jb.00159-25-s0004.xlsx Description of OMP profiles by strain.

ASM does not own the copyrights to Supplemental Material that may be linked to, or accessed through, an article. The authors have granted ASM a non-exclusive, world-wide license to publish the Supplemental Material files. Please contact the corresponding author directly for reuse.
