# Supplementary material for: Sequence variability of BamA and FadL candidate vaccinogens suggests divergent evolutionary paths of Treponema pallidum outer membrane proteins
Source: J Bacteriol. 2025 Jul 14;207(8):e00159-25. doi: 10.1128/jb.00159-25 (PMC12369335; doi:10.1128/jb.00159-25)
Supplement: Supplemental figures — Figures S1 to S3. [file jb.00159-25-s0001.pdf]

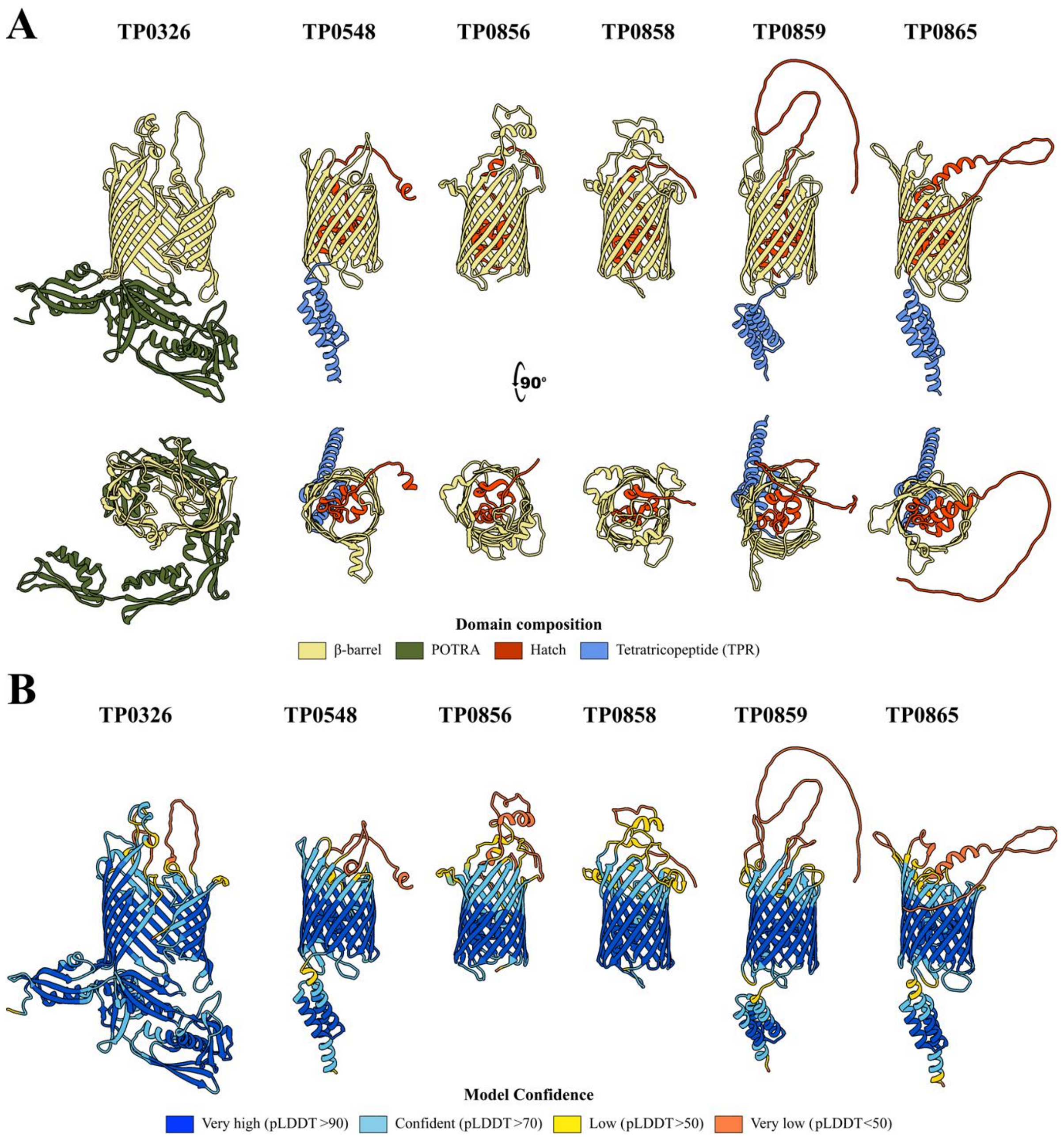

Supplementary Figure 1. AlphaFold3 models for TPA BamA and FadLs. (A) Protein models generated using AlphaFold3 are colored by domain composition. **(B)** The same models are colored according to a per-residue confidence metric (pLDDT) ranging from 1 to 100. Regions with pLDDT values >70 (light blue) and >90 (dark blue) denote high and very high confidence backbone predictions, respectively. All β-strand residues exhibited high confidence values at a minimum, supporting accurate delineation of the boundaries between β-strands and ECLs.

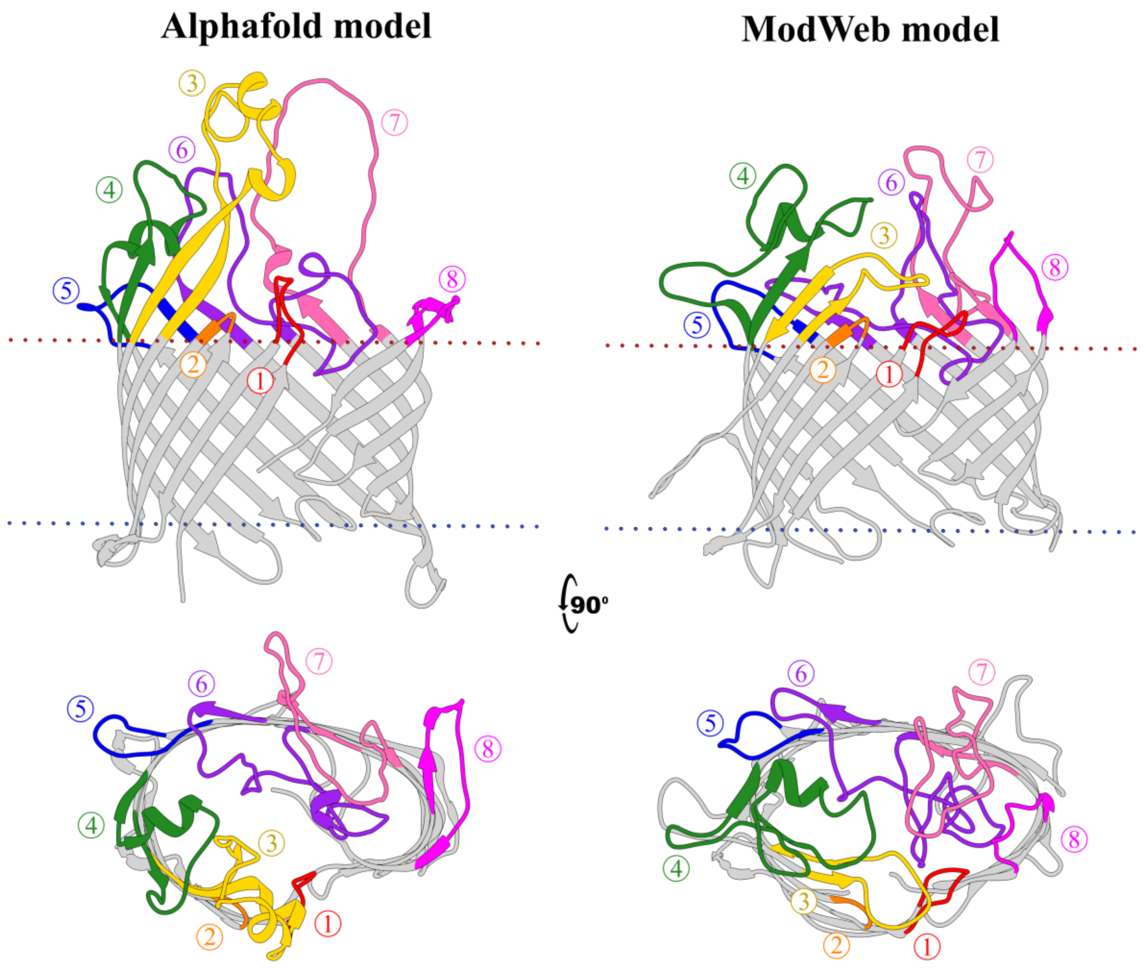

Supplementary Figure 2. Comparison of *T. pallidum* BamA models generated by AlphaFold and ModWeb. The model for BamA generated by AlphaFold3 predicts that ECL3 (yellow) is longer and ECL4 (green) is shorter than previously predicted by the ModWeb server using the solved structure of *Neisseria meningitidis* BamA ortholog as a template. ECL numbers are shown inside circles. Figures were generated using UCSF Chimera v1.1.

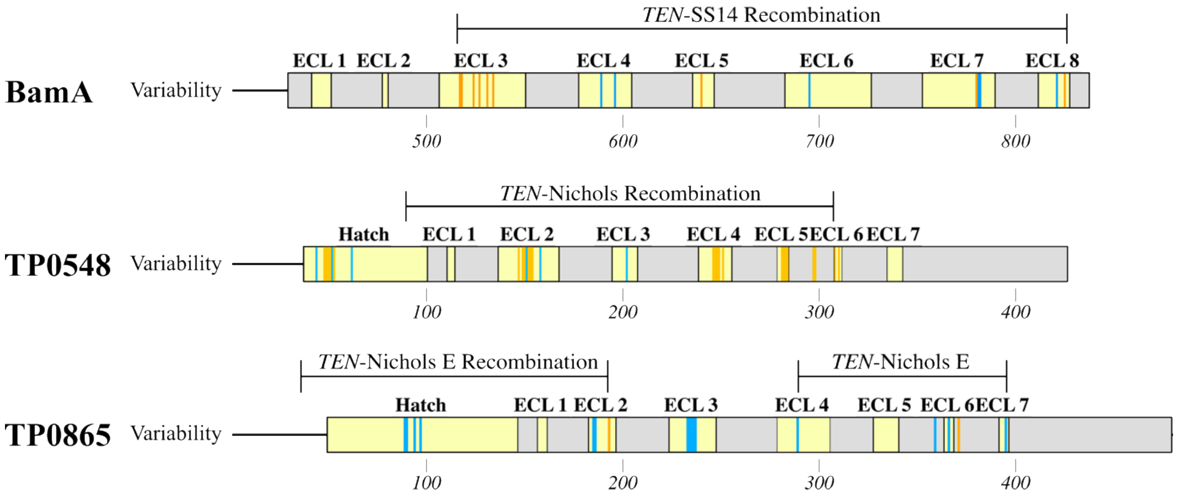

Supplementary Figure 3. Inter-subspecies recombination events previously reported for *TPA* BamA and FadL. (A) A recombinant event in a BamA region, corresponding to the nucleotide residues 347,027 to 347,956 of the Nichols reference genome, occurred between *TEN* and the SS14 clade. **(B)** In TP0548, a recombination between *TEN* and the Nichols clade occurred between residues 593,563 and 594,215. **(C)** Two previously reported recombination events in TP0865 involving *TEN/TPE* were identified here in all Nichols E strains (nucleotide residues 945,224 to 945,542 and 945,830 to 946,298). Mutation in the hypervariable ECL3 does not reside in any of the recombinant regions.
